# Supplementary material for: Mouth magnetoencephalography: A unique perspective on the human hippocampus
Source: Neuroimage. Author manuscript; Available in PMC 2021 Jul 7. (PMC8214102; doi:10.1016/j.neuroimage.2020.117443)
Supplement: Supplementary [file NIHMS1712046-supplement-Supplementary.docx]

**Mouth magnetoencephalography: Supplementary Figures**

***
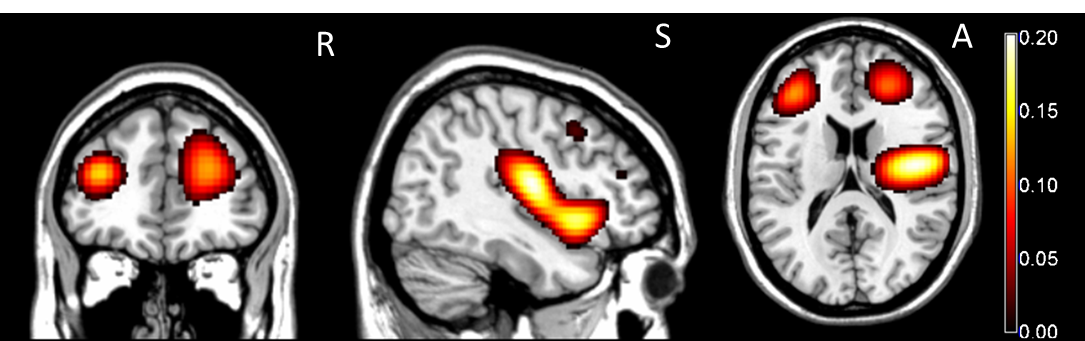
***

***Figure S1.*** *Mouth sensor coherence (1-4Hz) with the Beamformer reconstructed time series during the ‘Scene’ condition. Images are thresholded at FWE (p<.05). In the 1-4Hz band coherence was observed across the superior temporal gyrus, Insula and inferior frontal gyrus, and bilaterally in the middle frontal gyrus. No significant coherence with the hippocampus was observed in this band.*

*
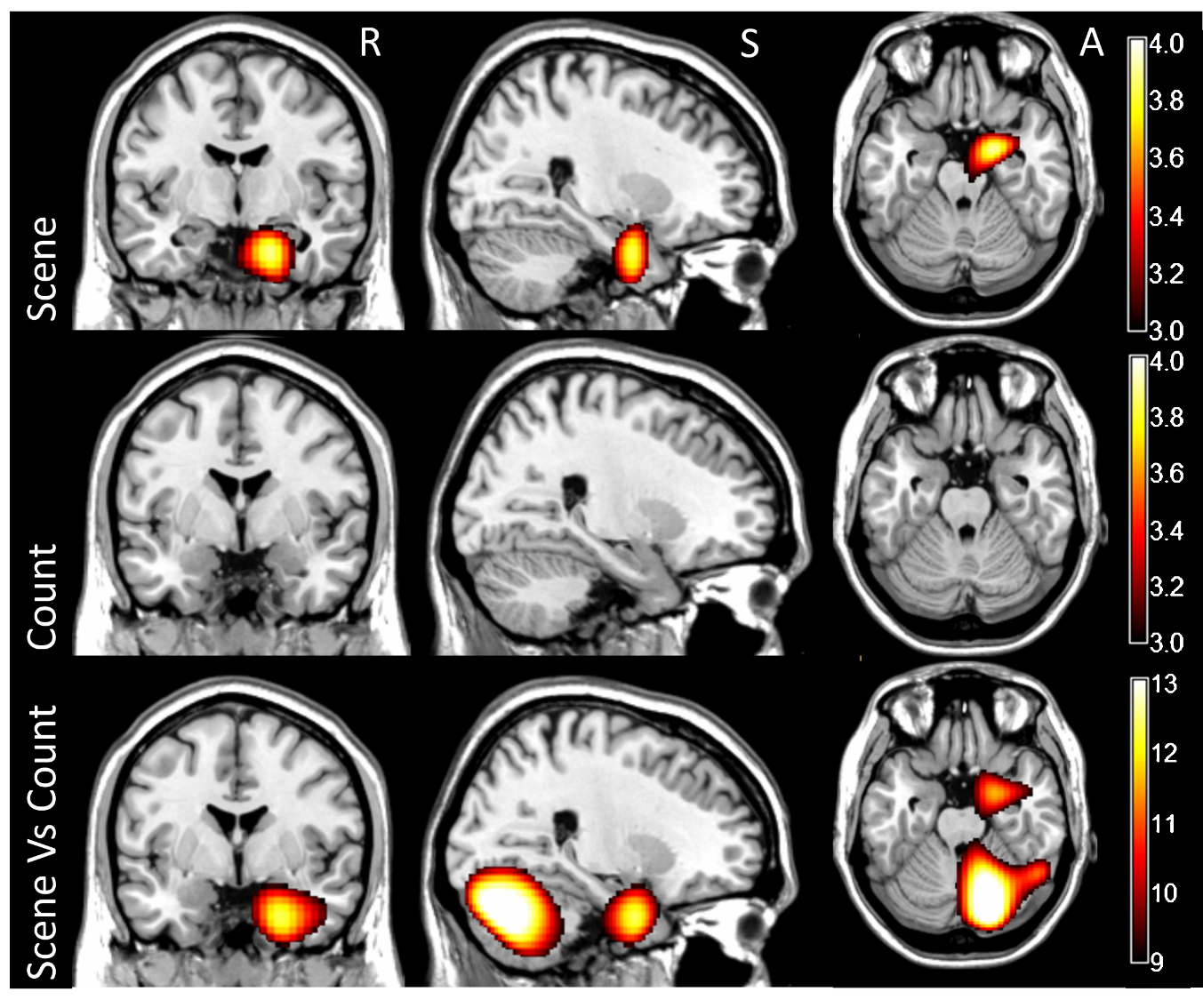
*

***Figure S2.*** *Beamformer power changes in the 4-8Hz band. The scene construction, counting and scene vs counting contrast are displayed on the MNI template at coordinate 24, -1,-24 using a statistical parametric map (F-statistic). The images are thresholded at F=3 for the scene and counting conditions and at F=9 for the contrast. In the scene Condition uncorrected power changes werre observed at one location covering the parahippocampal cortex, hippocampus and portions of the amygdala. Similar changes are seen in the contrast with the addition of a power change in the right cerebellum.*

***
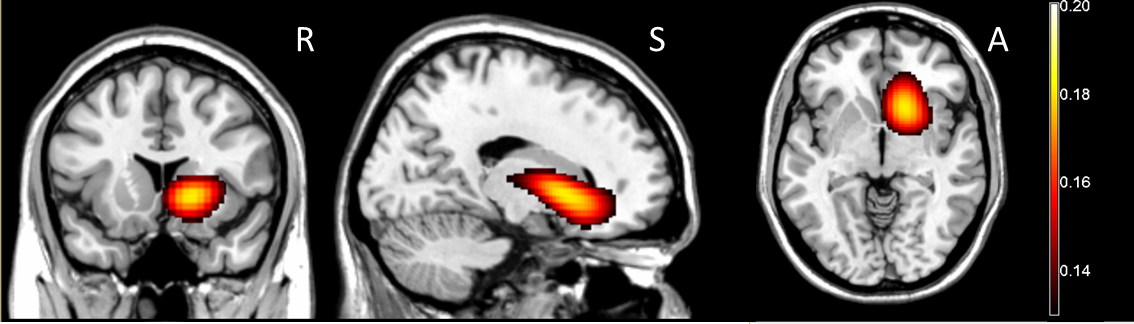
***
***Figure S3.*** *Mouth sensor coherence (4-8Hz) with the Beamformer reconstructed time series during the counting condition. Images are thresholded at FWE (p<.05). In the 4-8Hz band the global coherence peak was found covering the caudate, putamen and inferior portions of the frontal lobe. While not of primary interest to this study it is interesting to note that these areas are consistent with findings of a meta analyses of the brain areas involved in counting* ^1^*.*
